# Thunder-fire moxibustion for lumbar disc herniation: A systematic review and meta-analysis

**DOI:** 10.1097/MD.0000000000032270

**Published:** 2022-12-09

**Authors:** Jianye Chen, Zongxiu Luo, Mingda Liu, Fusheng Wang, Rui Zhou, Ying Wang, Yuyan Jia, Xukai Wang, Xiangyang Leng

**Affiliations:** a College of Traditional Chinese Medicine, Changchun University of Chinese Medicine, Changchun, Jilin Province, China; b The Affiliated Hospital of Changchun University of Chinese Medicine, Changchun, China.

**Keywords:** lumbar disc herniation (LDH), meta-analysis, meta-analysis, systematic review, thunder-fire moxibustion

## Abstract

Lumbar disc herniation (LDH) is a common degenerative disease that severely impacts the quality of life of patients. Thunder-fire moxibustion is an ancient Chinese medicine-based external therapeutic procedure that has been employed for pain relief until this day. The focus of our study was to demonstrate the effectiveness and safety of thunder-fire moxibustion in the treatment of LDH.

**Methods:**

The literature databases searched included the Cochrane Library, Web of Science, Springer, PubMed, Wanfang digital periodicals database, China national knowledge infrastructure, VIP, and Chinese biomedical literature database, and the search period was from database creation to March 2022. These include randomized controlled trials of Thunder–Fire moxibustion alone or in combination with other therapies for LDH. Two evaluators independently extracted data. We accessed the quality of inclusive studies through a Cochrane risk of bias tool. Meta-analyses were performed using Review Manager (Version 5.5). Data was analyzed using fixed-effects or random-effects models, depending on the heterogeneity test results.

**Results:**

The meta-analysis included 17 studies involving 1344 patients with LDH. The analysis results were as follows: compared with other therapies, the efficacy of thunder-fire moxibustion was statistically significant; the total effective rate (RR = 1.20; 95%CI [1.15, 1.26]; *P* < .00001), the Japanese orthopaedic association score (MD = 4.42; 95%CI [4.10, 4.73]; *P* < .00001), the pain score (SMD = -2.66; 95% CI [-3.39, -1.94]; *P* < .00001). Only 2 reported no adverse events in the included literature, and the remaining had no relevant records. The quality of the evidence in the 17 papers we examined was low or very low.

**Conclusion:**

Thunder–Fire moxibustion is effective in relieving discomfort in patients with LDH. It has significant clinical efficacy, but there is still a need for prospective, multicentre, large-sample randomized controlled trials to enhance the clinical evidence due to the quality of included studies and methodological limitations.

## 1. Introduction

Lumbar disc herniation (LDH) is a clinical degenerative spinal disease with corresponding clinical manifestations, which is caused by stimulation or compression of nerve roots due to rupture of annulus fibrosus and nucleus pulposus.^[1]^ The main manifestations of LDH are nerve compression symptoms such as pain in the lower back and legs, restricted movement, reduced muscle strength, and abnormal skin sensation.^[2]^ The prevalence of LDH is 2% to 3%,^[3]^ and 95% of patients have lesions involving the *L*4/5 and *L*5/*S*1 segments.^[4]^ Studies have shown that LDH is associated with age, obesity, smoking, exertion, and lumbar spine loading.^[5]^ Current treatments for LDH include conservative and surgical therapies.^[6]^ Due to the high cost and unknown risks associated with surgical treatment,^[7]^ most patients choose conventional treatment, including health education, physiotherapy and medication.^[8]^ Surgical treatment is not required when the patient has a short course of disease and the protrusions do not significantly compress the nerves, and there are no clinically significant symptoms of nerve damage.^[9]^ Non-operative treatment can relieve muscle spasm, nerve root adhesion and enlarge intervertebral space; It can also regulate endogenous pain-causing system, reduce the content of inflammatory factors in serum, and reduce stimulation to vascular receptors, thus relieving pain.^[10]^ Research has shown that 78.2% to 89.7% of patients have significant relief or resolution of clinical symptoms with conservative treatment.^[11]^ Although these conservative treatments have proven to have some effectiveness, they are not always effective and even have some side effects.

As a traditional therapy capable of preventing and treating diseases, moxibustion has been applied for thousands of years.^[12]^ It has been widely used as early as the Spring and Autumn and Warring States period.^[13]^ Thunder–Fire Moxibustion is a new moxibustion method that improves the original formula and usage. It is based on the meridian theory of Chinese medicine, combining the idea of acupuncture and moxibustion with the action of a drug, using moxa and herbs to make medicinal moxa strips.^[14]^ Compared to ordinary moxa strips, it has a thicker form, stronger potency, more substantial penetration, and a wider area of action.^[15]^ Thunder-fire moxibustion is commonly utilized in China to treat various pain-related conditions, including ENT (ear-nose-throat), bone and joint, gynecological, and internal diseases.^[16–22]^ Thunder–Fire moxibustion uses the heat generated by the burning of the drug, infrared radiation, and medicinal and physical factors to reach the treatment area through the meridian and acupuncture point sensory transmission.^[23]^ The chemical substances produced by it promote the material exchange of tissue cells, so it has the functions of warming the meridians, promoting blood circulation and removing blood stasis, dispelling wind and dispelling cold.^[24]^ It is fully recognized by patients in clinical operations because of its simple usage, painless treatment, and few adverse reactions.^[23]^

Although the benefits of Thunder–Fire moxibustion for LDH have been widely reported, only individual studies have been conducted. None have systematically evaluated the use of thunder-fire moxibustion alone or in combination with other therapies for LDH. Therefore, this paper analyses the efficacy and safety of Thunder–Fire moxibustion in treating LDH to provide Medical evidence-based for the cure of LDH in Chinese medicine.

## 2. Methods

### 2.1. Registration

The International Prospective Register of Systematic Reviews recorded this work (CRD42022312569). We write this paper based on the preferred reporting items for systematic reviews and meta-analyses guide.^[25]^

### 2.2. Eligibility criteria

#### 2.2.1. Types of study.

We included all randomized controlled trials of Thunder–Fire moxibustion for LDH. There were no restrictions on the country and language.

#### 2.2.2. Types of participants.

Adults diagnosed with LDH are not restricted by gender, ethnicity, or region. However, studies with specific or systemic diseases will be excluded (spinal tumors, caudal equine syndrome, lumbar spondylolisthesis, fractures, severe osteoporosis, and pregnancy patients).

#### 2.2.3. Types of intervention.

The intervention group was treated with either single thunder-fire moxibustion or in combination with other therapies for LDH. Still, thunder-fire moxibustion was required to be the only treatment variable. In contrast, the control group was treated with other therapies other than Thunder–Fire moxibustion, such as acupuncture, massage, western medicine, fire cupping, etc.

#### 2.2.4. Types of outcomes.

We selected the total effective rate, Japanese orthopaedic association (JOA) score, pain score, Oswestry disability index (ODI), and 36-item short-form health survey (SF-36) as outcome indicators. The overall effectiveness of each study included relief of signs and symptoms of low back pain, lower limb numbness, lower limb motor function, and lumbar spine range of motion. Although the definition of “overall effectiveness” differs from study to study, all refer to remission of disease and recovery of daily living and working abilities. The JOA score for lower back pain consists of 4 components: subjective symptoms (lower back pain, leg pain, gait), clinical signs (straight leg raising, sensory disturbance, motor disturbance), limitation of daily activities and bladder function, with a total score of 29 points, the higher the score, the better the patient’s lumbar function.^[26]^ Pain scores include the Numerical rating scale or the visual analogue scale (VAS), with lower scores indicating less pain.^[27]^ The ODI assesses the patient’s return to socially active functioning, with lower scores indicating less severe disease.^[28]^ The SF-36 assesses the patient’s quality of life before and after treatment, with higher scores indicating better quality of life.^[29]^

### 2.3. Data sources

We searched 8 databases in English and Chinese, including Cochrane Library, Web of Science, Springer, PubMed, Wanfang digital periodicals database, China national knowledge infrastructure database, Chinese biological medicine database, and VIP Database. These databases were searched from their inception to March 2022.

### 2.4. Search strategy

The search terms: Intervertebral Disc Displacement (e.g., Intervertebral Disc Displacement, Intervertebral Disc Degeneration, Lumbar Disc Herniation) and Tunder-fire Moxibustion (e.g., Tunder–Fire Moxibustion, Leihou Moxibustion). These search terms are suitable for searching these databases. Table 1 shows how PubMed is searched.

**Table 1:** Search strategy.

Number	Search terms
01	Intervertebral Disc Displacement
02	Intervertebral Disc Degeneration
03	Lumbar Disc Herniation
04	Or 01–03
05	Thunder-fire moxibustion
06	Leihuo moxibustion
07	Or 05–06
08	Randomized controlled trial
09	Randomized
10	RCT
11	Or 08–10
12	04 and 07 and 11

### 2.5. Data collection and analysis

#### 2.5.1. Research selection.

Two trained literature screeners searched the database to obtain the required literature according to the proposed search formula. In the event of a dispute, a third researcher engaged in the conversation and negotiated the decision. Firstly, they imported the selected literature into NoteExpress X9 and removed duplicates. Secondly, the 2 researchers (Luo Zongxiu and Liu Mingda) read the titles and abstracts and then excluded the literature that did not fit the study. Finally, the full text of the literature that matched the study was downloaded and read in detail.

#### 2.5.2. Data extraction.

Two researchers extracted relevant information from the included literature and managed the registration using an Excel sheet. The data extracted included the name of the researcher, year of publication, the sample size of the treatment and control groups, interventions, duration of treatment, and outcome indicators. If important information was missing, we called the author to get information about the research.

#### 2.5.3. The assessment for risk of bias.

Two researchers used Cochrane’s risk of bias (ROB)^[30]^ to assess the risk of bias. The risk of bias assessment tool included 7 items: random sequence generation, allocation concealment, blinding of researchers, subject and outcome assessment, incomplete outcome information, selective result reporting, and other sources of bias. A third researcher was involved in the discussion and consultation in case of disagreement.

#### 2.5.4. Data analysis and synthesis.

Meta-analysis of the data was performed using Review Manager (Version 5.5). The results of dichotomous variables were expressed as relative risk (RR), mean difference (MD) or standardized mean difference (SMD) for continuous variables, all with 95% confidence intervals (CI). We chose the *χ*^2^ test and the *I*^2^ test to determine heterogeneity. When *P* ≥ .1, *I*^2^ ≤ 50%, we consider the data homogeneous and select the fixed effect model. If *P* < .1, *I*^2^ > 50%, the data were supposed to be heterogeneous, and the source of heterogeneity was speculated using sensitivity analysis or subgroup analysis. Descriptive analysis or the random-effects model is chosen when we cannot find the cause of the heterogeneity.

#### 2.5.5. Level of evidence.

We analyzed the evidence level with the Grading of Recommendations, Assessment, Development, and Evaluation (GRADE).^[31]^ GRADE’s 5 factors may reduce the quality of evidence in systematic interventional reviews: risk of bias, inconsistency, imprecision, indirectness, and publication bias. The quality of the evidence was categorized into the following 4 levels: high quality, moderate quality, low quality, and very low quality, representing the strength of the evidence.

### 2.6. Search results.

**Figure 1.**
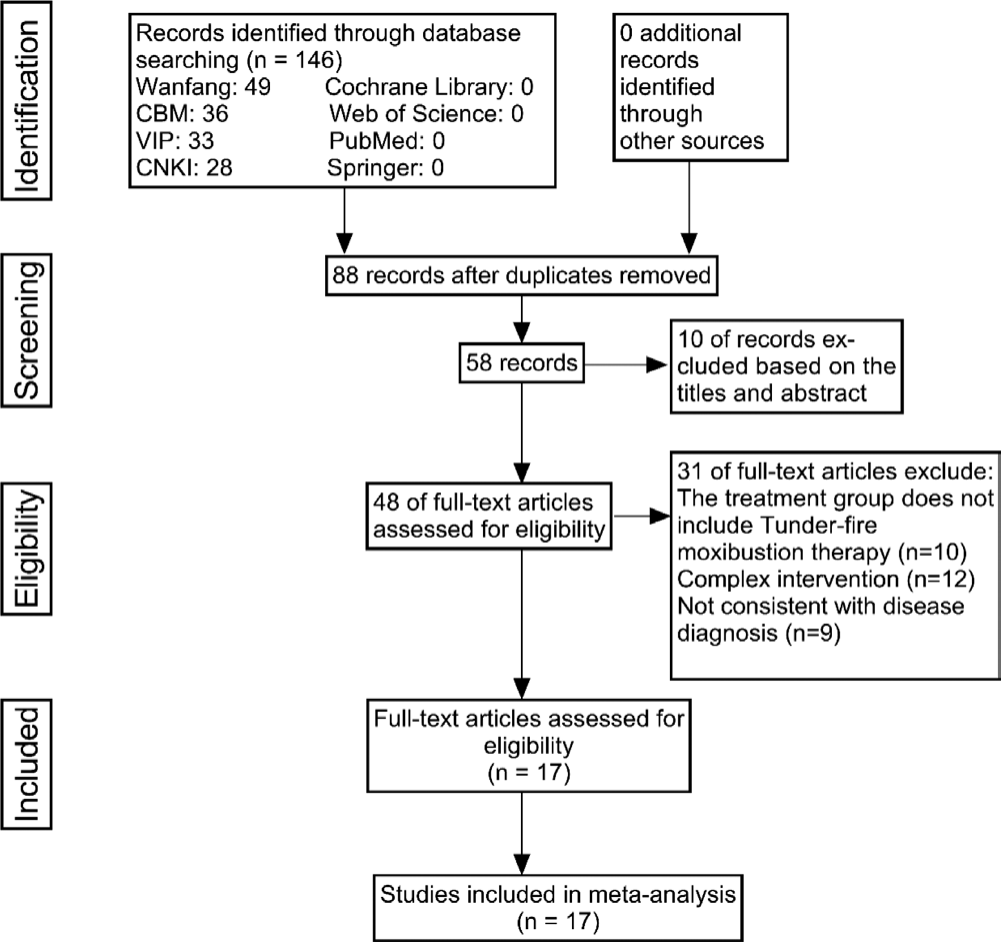
Flow diagram of studies identified.

One hundred forty-six trials were searched in the database, 28 from China national knowledge infrastructure, 33 from VIP, 49 from Wanfang digital periodicals database, 36 from Chinese biomedical literature database, 0 from Cochrane Library, 0 from Web of Science, 0 from Springer, and 0 from PubMed. Eighty-eight papers were excluded due to duplication. After reading the titles and abstracts, 10 irrelevant trials were excluded. Forty-eight articles that remained were read in full. After further reading, 10 papers were excluded because the treatment group did not include thunder-fire moxibustion. Twelve were excluded for complex interventions. Nine were excluded from the disease diagnosis. The last 17 studies were included in the meta-analysis (Fig. 1).

### 2.7. Study characteristics

The total sample size of the 17 included papers was 1344 cases, including 673 cases in the trial group and 671 cases in the control group. These studies described comparable baseline information between the treatment and control groups, with no statistically significant difference (*P* > .05). Five studies^[32–36]^ reported treatment duration less than or equal to 10 days, 11 studies^[37–47]^ reported treatment durations more than 10 days, and 1 study^[48]^ did not report treatment duration. Fifteen studies^[32,33,35,37–48]^ used the total effective rate, 8 studies^[33,34,36,38,39,43,44,46]^ used the JOA score, 6 studies^[33,36,38,43,44,46]^ used the pain score, 2 studies^[32,35]^ used the ODI,and 1 study^[39]^ used the SF-36. The clinical characteristics of the specific literature are shown in Table 2.

**Table 2:** Characteristics of the included studies.

First author (yr)	Participants		Sample size (male/female)	Mean age (range) (year)	Intervention		Duration	Outcome	Moxibustion acupoint
	Treated group	Control group			Treated group	Control group			
Cai 2014^[37]^	39	39	T: 20/19	T:(30–65)	a + e	e	30d, 10 min/d	①	EX-B2,B*L*-23,BL-45 GB-30,B*L*-40,GB-39
			C: 17/22	C:(27–65)					
Chen 2020^[38]^	30	30	T: 19/11	T:(41.6 ± 6.7) (28–65) C:(42.4 ± 6.5) (30–64)	a + b + d	b + d	20d, 30-45min/d	①②③	ST-41,GB-39,GB-30 GB-34
			C: 17/13						
Chen 2021^[39]^	60	60	NR	NR	a + c	c	20d, 10 min/d	①②⑤	ashi point
Ding 2015^[40]^	30	30	NR	NR	a	e	14d, 25 min/d	①	BL-40 ST-36
Jiang 2019^[41]^	30	30	T: 16/14	T:(45.5 ± 3.3) (26–65)	a + c	c	24 d, 15–20min/d	①	GB-30,B*L*-36,BL-23 BL-40,G*V*-3, ashi point
			C: 17/13	C: (46.0 ± 3.5) (26–66)					
Jin 2014^[42]^	30	30	T: 17/13	T:(45.78 ± 5.52) (22–60)	a + d	d	20 d, 15–20min/d	①	BL-23 GB-30 BL-40
			C: 16/14	C:(45.23 ± 6.25) (22–65)					
Liu 2021^[43]^	30	30	T: 20/10	T:(52.42 ± 3.17) (40–61) C:(52.38 ± 3.25) (42–60)	a + e	e	24 d, 15 min/d	①②③	BL-43
			C: 21/9						
Mao 2016^[44]^	33	32	T: 16/17	T:(40.1 ± 9.40) C: (39.2 ± 9.1)	a + b	b	14d, 30–60min/d	①②③	BL-23 GB-30 BL-40
			C: 21/14						
Su 2013^[45]^	26	26	NR	T:(24–62)	a + b	b	20 d, ≥10 min/d	①	BL-23, GV-4, BL-31 GV-3, BL-25 BL-32 BL-33, BL-34
				C:(24–62)					
Wang 2018^[32]^	52	51	T: 28/24	T:(52.76 ± 8.89)	a + e	e	10 d, 60 min/d	①④	GB-30, BL-40
			C: 26/25						
				C:(53.64 ± 8.45)					
Xu 2016^[33]^	60	60	T: 39/21	T:(43.10 ± 5.30) (30–64) C:(42.70 ± 5.30) (28–65)	a + d	d	3d, 40–60min/d	①②③	EX-B2,B*L*-40
			C: 37/23						
Yang 2014^[46]^	64	64	T: 38/26	T:(58.2 ± 12.4) (35–72) C:(57.9 ± 11.6) (32–70)	a + b	b	21d, 20 min/d	①	BL-28, BL-23
			C: 35/29						
Yao 2014^[48]^	43	43	NR	T:(18–83)	a + d	d	NR 20–30min/d	①	ashi point
				C:(18–83)					
Yang 2015^[34]^	30	30	T: 10/20	T:(39.30 ± 16.14) C:(38.40 ± 15.52)	a + c	c	10d, 50–60min/d	②	GB-30,B*L*-40
			C: 11/19						
Yang 2018^[47]^	26	26	NR	T:(24–62)	a + b	b	20 d, 10 min/d	①	BL-23, GV-4 GV-3, BL-25
				C:(24–62)					
Zheng 2019^[35]^	50	50	T: 30/20	T:(54.49 ± 8.19) (35–74) C:(54.26 ± 8.24) (34–72)	a + b	b	10 d, 60 min/d	①②③④	BL-23, GB-30, BL-40,G*V*-3
			C: 29/21						
Zou 2021^[36]^	40	40	T: 22/18	T:(22–54) C:(23–57)	a + b	b	10 d, 50–60min/d	②③	GB-30,B*L*-40
			C: 25/15						

(T, experimental group; C, control group; NR, not reported; (a), Thunder–fire moxibustion; (b), manipulative therapeutics; (c), acupuncture; (d), routine nursing; (e), medicine; ①total effective rate; ②JOA score; ③pain score; ④ODI; ⑤SF-36).

### 2.8. Risk of bias assessments

**Figure 2.**
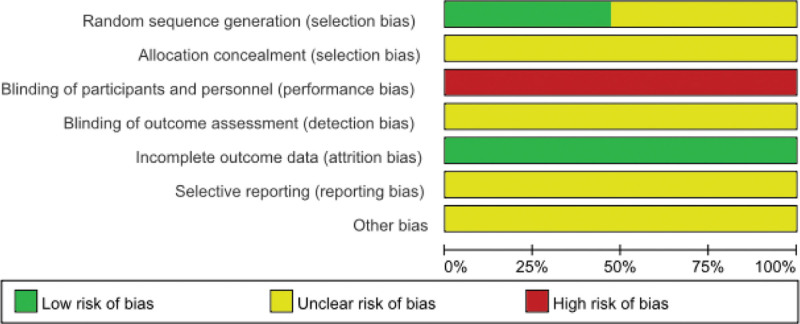
Risk of bias of the included studies.

**Figure 3.**
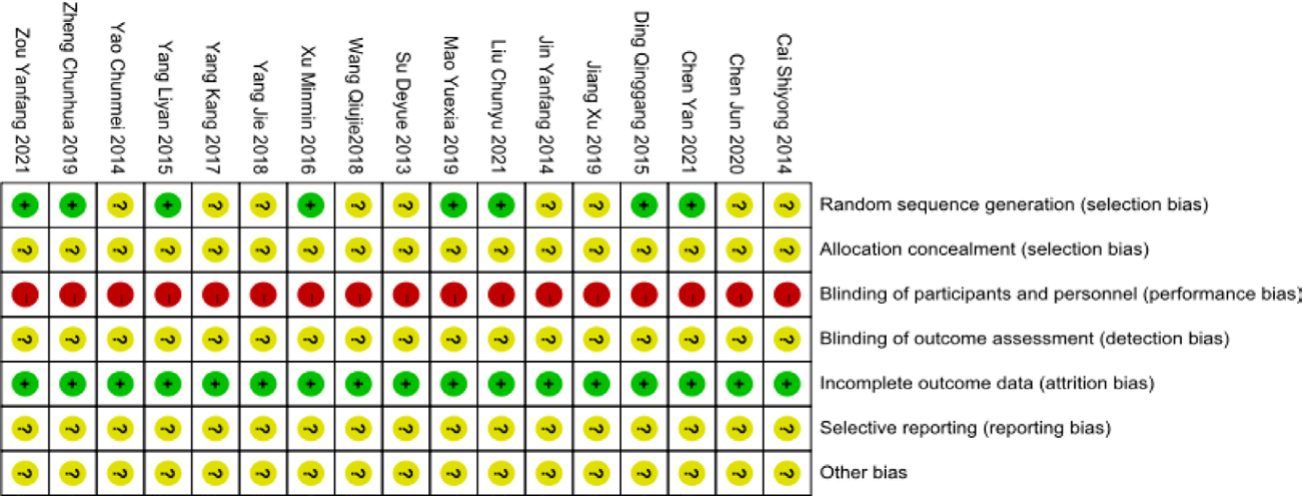
Risk of bias summary.

Eight studies^[33–36,39,40,43,44]^ utilized specific randomization methods, whilst others simply stated “random.” All studies did not record whether concealment was assigned. Due to the specific nature of thunder-fire moxibustion treatment, all trials could not be blinded. Data completeness was good in all studies. Selective reporting was classified as unclear as there were not too many outcome indicator to determine selective reporting. Other risks of bias were known as unclear because all randomized controlled trials were not registered (Figs. 2 and 3).

## 3. Outcomes

### 3.1. Total effective rate

**Figure 4.**
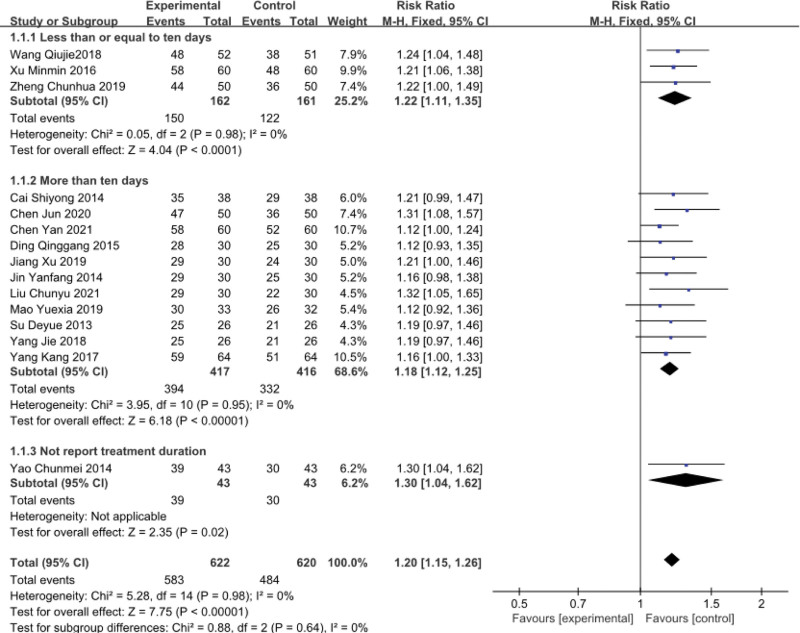
Forest plots of total effective rate.

In the 17 studies, 15^[32–34,37–48]^ reported the total effective rate. Three studies found total effective rates of less than or equal to 10 days,^[32,33,35]^ eleven studies reported total effective rates of more than 10 days,^[37–47]^ and 1 study did not expound treatment duration.^[48]^ The results showed better clinical effect in the treatment group than in the control group, and the differences were statistically significant (RR = 1.20; 95% CI [1.15, 1.26]; *P* < .00001; *I*^2^ = 0). There was no heterogeneity in the total effective rate (less than or equal to 10 days) (RR = 1.22; 95% CI [1.11, 1.35]; *P* < .00001; *I*^2^ = 0). The total effective rate (more than 10 days) had statistical significance (RR = 1.18; 95% CI [1.12, 1.25]; *P* < .00001; *I*^2^ = 0) with no heterogeneity. The total effective rate (not reported treatment duration) had statistical significance (RR = 1.30; 95% CI [1.04, 1.62]; *P* = .02). According to the findings, thunder-fire moxibustion had a better effect than the control group (Fig. 4).

### 3.2. JOA score

**Figure 5.**
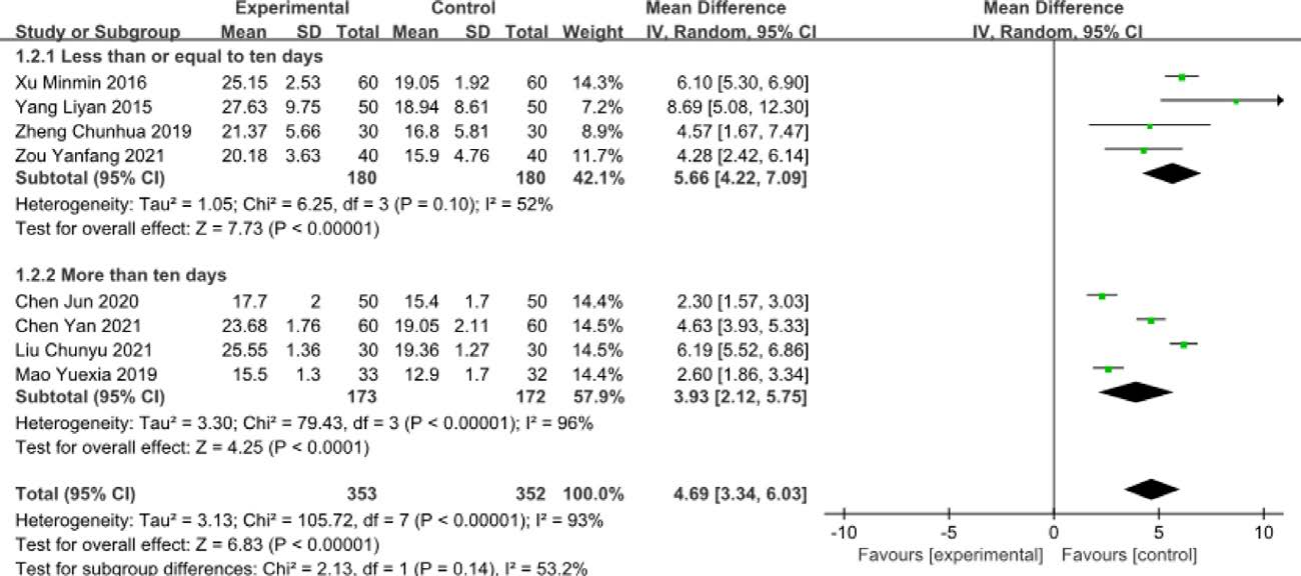
Forest plots of JOA score. JOA = Japanese orthopaedic association.

Eight trials^[33–36,38,39,43,44]^ evaluated lumbar spine function by using JOA score. The result showed that MD = 4.69 (*P* < .00001), indicating that Thunder–Fire moxibustion was more effective than the control group. However, there was substantial heterogeneity (*I*^2 ^= 93%). Because of the heterogeneity, we used a random-effects model and did a subgroup analysis based on treatment duration. JOA score (less than or equal to 10 days) had statistical significance (MD = 5.66; 95% CI [4.22,7.09]; *P* < .00001; *I*^2^ = 52%) with heterogeneity. JOA score (more than 10 days) had statistical significance (MD = 3.93; 95% CI [2.12,5.75]; *P* < .00001; *I*^2^ = 96%) with heterogeneity (Fig. 5).

### 3.3. Pain score

**Figure 6.**
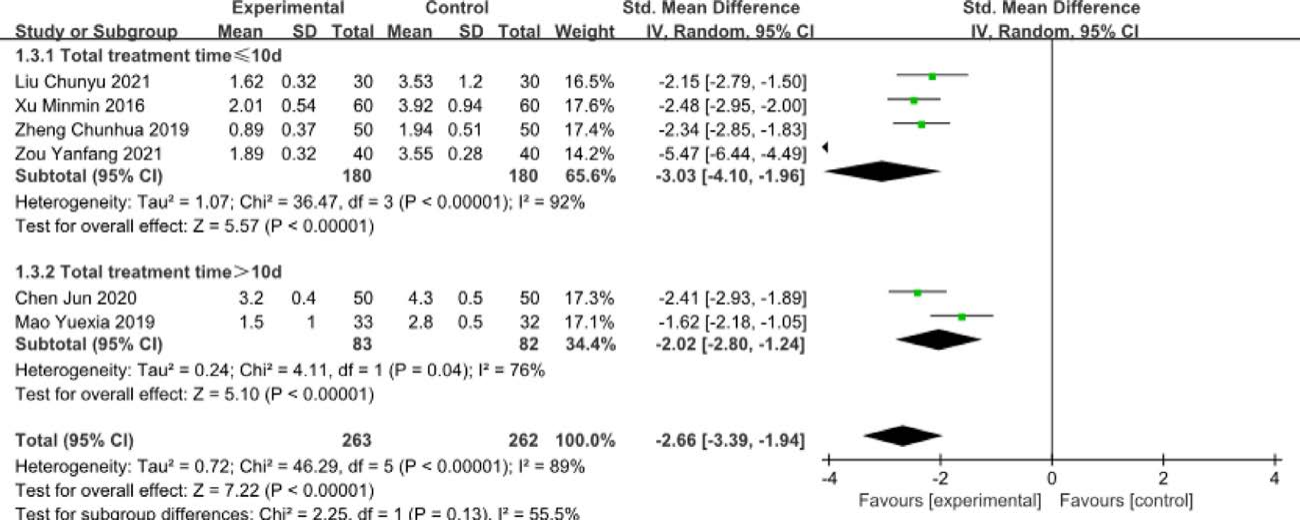
Forest plots of pain score.

Pain scores were reported in 6 trials, 1 used numeric rating scale, and the others used VAS. The worse situation, the higher the score. The results showed that Thunder–Fire moxibustion could significantly reduce pain (SMD = -2.66; 95%CI [-3.39, -1.94]; *P* < .00001), which is more effective than the control group, but there is obvious heterogeneity (*I*^2 = ^89%). To find the causes of heterogeneity, we used a sensitivity analysis. After removing the study of Zou Yanfang, the heterogeneity dropped to 36%. We speculate that this study has a longer single treatment time than other studies, which may be the main reason. Pain score (total treatment time ≤ 10d) was statistically significant (SMD = -3.03; 95%CI [-4.10, -1.96]; *P* < .00001; *I*^2^ = 92%). Pain score (total treatment time > 10 days) was statistically significant (SMD = -2.02; 95%CI [-2.80, -1.24]; *P* < .00001; *I*^2^ = 76%) (Fig. 6).

### 3.4. ODI score

Two studies^[32,35]^ used the ODI score to evaluate the recovery of social function. The lower the score, the better the patient’s social function. One study^[32]^ did not provide specific values but only recorded that the experimental group was treated with thunder-fire moxibustion based on the conventional treatment of the control group. The results showed that the ODI score of the experimental group was significantly lower than that of the control group (*P* < .05). One study^[35]^ reported the comparison of thunder-fire moxibustion combined with manipulative therapeutics and manipulative therapeutics alone, and the results showed that the experimental group was more effective than the control group (MD = -8.19; 95%CI [-8.78, -7.60]; *P* < .00001).

### 3.5. SF-36 scale

One trial^[39]^ used the SF-36 scale to evaluate the quality of life. Based on acupuncture combined with thunder-fire moxibustion, the results showed that the experimental group could effectively improve the quality of life (MD = 4.63; 95%CI [3.93, 5.33].

### 3.6. Adverse events

In this meta-analysis, 2 studies^[34,36]^ reported no significant adverse events, whereas other studies^[32,33,35,37–48]^ did not analyze adverse events.

### 3.7. Choice of acupoints

We summarized the use of acupoints in 17 pieces of literature. Three studies^[32,34,36]^ used the same combination of acupoints, and the remaining studies^[33,35,37–48]^ included different acupoints. GB30 (10 studies,^[32,34–40,42,44]^ 58.8%) had the highest frequency of use, followed by BL40 (9 studies ^[32–37,41,42,44]^, 52.9%), BL23 (8 studies,^[35,37,41,42,44–47]^ 47%), GV3 (4 studies,^[35,41,45,47]^ 23.5%). Other acupoints appeared less frequently (Table 3).

**Table 3:** Characteristics of the included studies.

Order	Acupoints	Frequency (%, N = 17)
1	GB30	10 (58.8%)
2	BL40	9 (52.9%)
3	BL23	8 (47%)
4	GV3	4 (23.5%)
5	BL25, Ashi point	3 (17.6%)
6	EX-B2, GV4, GB39	2 (11.7%)
7	ST36, BL28, BL3134, ST41, GB34, BL36, BL43	1 (5.8%)

### 3.8. Publication bias

**Figure 7.**
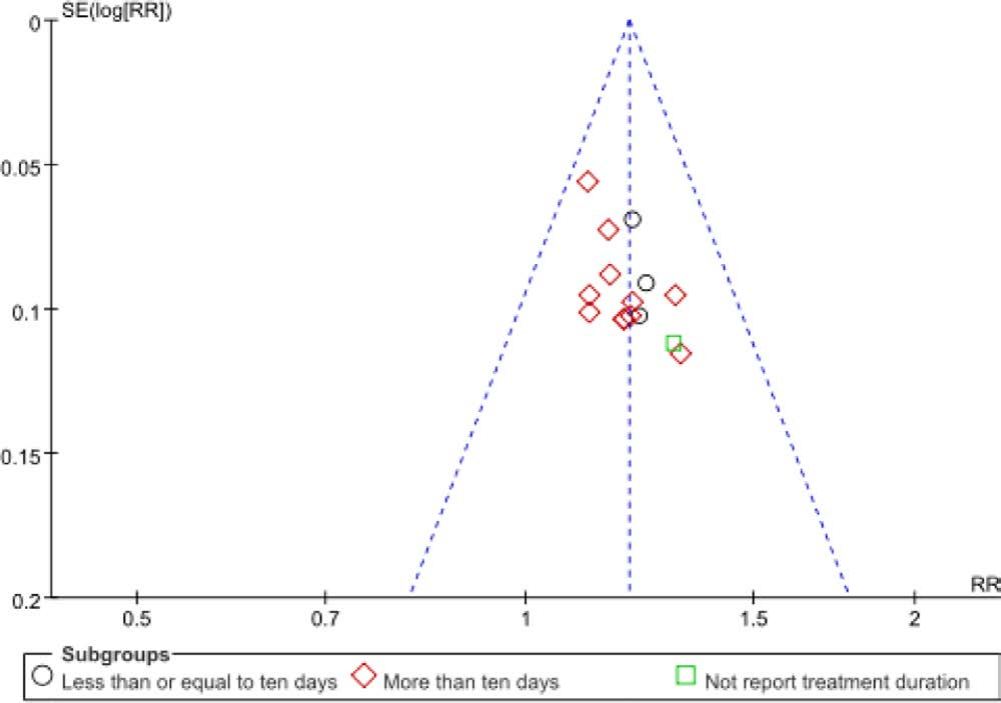
Funnel plot of the publication bias.

The total efficacy rate of the 15 randomized controlled trials was plotted using a funnel plot. We can find that the funnel plot of the total effective rate is not symmetrical (Fig. 7), indicating that this study may have publication bias. The analysis shows that there may be reasons for inconsistent intervention methods among treatment groups and differences in the methodological quality of included literature. To avoid bias, it is important to improve the quality of the study and increase the sample size of the study.

### 3.9. Level of evidence

GRADE was used to evaluate the evidence’s quality. The quality of the studies’ major outcomes (the total effective rate, JOA score, and pain score) was assessed. The following 5 factors can reduce the level of evidence: risk of bias, inconsistency, imprecision, indirectness, and publication bias. All included studies reported outcomes directly. Some studies did not state the specific randomization method. None of the studies mentioned the specific implementation process of blinding and allocation concealment. It was not possible to judge the selectively reported outcomes. There may be some heterogeneity in JOA and pain scores depending on the duration of treatment. From the efficient funnel plot analysis, the asymmetry on both sides indicates the existence of publication bias. All of the above contribute to the lowered level of evidence. The quality of evidence for all comparisons was low or very low (Table 4).

**Table 4:** GRADE certainty grading evaluation.

Certainty assessment								Summary of results				Certainty
NO. of studies	Type of Study	Risk of bias	Inconsistency	Indirectness	Imprecision	Publication bias	Other	Number of cases		95%CI		
								T	C	Binary variable	Continuous variable	
The total effective rate												
15	RCT	Serious1	No serious	No serious	No serious	Possible3	None	622	620	RR = 1.20 [1.15,1.26]		⊕⊕○○ Low
JOA score												
8	RCT	Serious1	Very serious2	No serious	No serious	Possible3	None	353	352		MD = 4.69 [3.34,6.03]	⊕○○○ Very low
Pain score												
6	RCT	Serious1	Very serious2	No serious	No serious	Possible3	None	263	262		SMD = -2.66 [-3.39, -1.94]	⊕○○○ Very low

(1) There is a risk of bias in allocation concealment and blinding. (2) Considerable heterogeneity. (3) All included papers are Chinese.

JOA = Japanese orthopaedic association.

## 4. Discussion

Until now, no researchers have discussed a systematic review and meta-analysis of thunder-fire moxibustion in the treatment of LDH. This is the first study to evaluate the efficacy and safety of Thunder–Fire moxibustion in patients with LDH. This study found that Thunder–Fire moxibustion was superior to the control group in improving the total effective rate, JOA score, pain score, ODI score, and SF-36 scale, with statistically significant differences. Thunder–Fire moxibustion can alleviate low back pain, lower extremity numbness, and other symptoms, but there is heterogeneity in tests of JOA score and pain score. By observing the subgroup analysis of the forest plot, we found that it may be related to the treatment time and the duration of the disease. Still, this conclusion needs to be verified by further clinical trials or network meta-analyses. Because the intervention measures (acupuncture, massage, traditional Chinese medicine manipulation, medicine, and other treatment methods) are too complicated, it is difficult to objectively reflect the therapeutic effect of Thunder–Fire moxibustion in the treatment of LDH.

Only 2 of the studies reported no adverse events in terms of safety summaries, and the remaining 15 were not recorded. Therefore, there isn’t enough evidence to prove the safety of Thunder–Fire moxibustion for LDH.

In this meta-analysis, although some data show that Thunder–Fire moxibustion can improve the symptoms of LDH patients, many factors affect the efficacy of Thunder–Fire moxibustion. The quality of the included literature is low. The sample size is small. The production technology and level of Thunder–Fire moxibustion are different. The evaluation index is subjective. As a result of the above, getting high-quality evidence is difficult,^[49]^ which makes it hard to explain the efficacy of Thunder–Fire moxibustion in treating LDH.

Research^[50]^ shows that thunder-fire moxibustion is extensively used for pain caused by various diseases, which can successfully prolong alleviation, reduce pain duration, minimize recurrence rate, and prevent harmful effects. Thunder–Fire moxibustion has been found to minimize inflammatory exudation, decrease the release of inflammatory factors including Tumor Necrosis Factor (TNF) and interleukin-1 (IL1), and promote the absorption of inflammatory exudates. Therefore, it has anti-inflammatory, analgesic, and immune-enhancing effects.^[51,52]^ It’s essential to look into Thunder–Fire moxibustion’s mechanism, effectiveness, and safety.

This study has the following shortcomings. The included literature did not describe whether allocation concealment was implemented or not. If it was not implemented or was not implemented sufficiently, the concealed grouping would exaggerate the efficacy. Outcome measures are subjective. Due to the specificity of the intervention, blinding was not possible. The operation of Thunder–Fire moxibustion in each study is different: treatment time, differences in acupoints, control of Thunder–Fire moxibustion temperature and distance, etc. The above factors will affect the effect. Although we searched databases in both Chinese and English, the final included articles were Chinese, which may lead to publication bias. These studies did not address follow-up, resulting in an inability to evaluate long-term efficacy accurately. This study graded the evidence for a single outcome measure using the GRADE system tool. The quality of the evidence for the included studies was low or very low, indicating that the real situation may be significantly different from the conclusions of the systematic review. Because of the above shortcomings, we should cautiously explain the efficacy of thunder-fire moxibustion for LDH.

We suggest the following strategies for future researchers. They should use internationally agreed diagnostic criteria and report the specific methods of random sequence generation and allocation concealment in detail. Due to the highly subjective nature of pain scales, studies have suggested that a reduction of 20% or more in the VAS score is more meaningful.^[53]^ Investigators should follow up with patients regularly and strengthen monitoring and reporting of adverse events. In conclusion, we should further enhance the authenticity and reliability of the research results to guide the clinical practice better.

## 5. Conclusion

This study shows that thunder-fire moxibustion can improve patients’ symptoms in the treatment of LDH. At the same time, it has the advantages of convenient operation, convenient nursing and fewer adverse reactions. However, based on the quality of the current clinical trials, we cannot conclude the efficacy of Thunder–Fire moxibustion in treating LDH. To provide higher-level evidence support for the treatment of LDH with Thunder–Fire moxibustion, it is also necessary to conduct more high-quality randomized controlled studies and identify more complete outcome indicators.

## Acknowledgments

TCM Clinical Research Center for Bone diseases of Jilin Province (Grant No. 20180623048TC). Research on medication rules and clinical experience of Professor Liu Bailing, a master of traditional Chinese med icine based on data mining (subject of Jilin Provincial Administration of Traditional Chinese Medicine) (No. 2022153).

## Author contributions

**Conceptualization:** Zongxiu Luo, Fusheng Wang.

**Data curation:** Zongxiu Luo, Mingda Liu.

**Formal analysis:** Jianye Chen, Yuyan Jia.

**Investigation:** Mingda Liu, Rui Zhou, Ying Wang.

**Methodology:** Yuyan Jia, Fusheng Wang.

**Software:** Jianye Chen, Zongxiu Luo, Mingda Liu.

**Writing – original draft:** Rui Zhou, Ying Wang, Jianye Chen.

**Writing – review & editing:** Xukai Wang, Xiangyang Leng.
